# Missed Diagnoses and Health Problems in Adults With Prader-Willi Syndrome: Recommendations for Screening and Treatment

**DOI:** 10.1210/clinem/dgaa621

**Published:** 2020-09-02

**Authors:** Karlijn Pellikaan, Anna G W Rosenberg, Anja A Kattentidt-Mouravieva, Rogier Kersseboom, Anja G Bos-Roubos, José M C Veen-Roelofs, Nina van Wieringen, Franciska M E Hoekstra, Sjoerd A A van den Berg, Aart Jan van der Lely, Laura C G de Graaff

**Affiliations:** 1 Internal Medicine, Division of Endocrinology, Erasmus MC, University Medical Center Rotterdam, GD Rotterdam, Netherlands; 2 Stichting Zuidwester, LB Middelharnis, Netherlands; 3 Vincent van Gogh, Center of Excellence for Neuropsychiatry, DN Venray, Netherlands; 4 ‘s Heeren Loo, Care Providing Agency, SC Wekerom, Netherlands; 5 Department of Physical Therapy, Erasmus MC, University Medical Center Rotterdam, GD Rotterdam, Netherlands; 6 Department of Internal Medicine, Reinier de Graaf Hospital, AD Delft, Netherlands; 7 Department of Clinical Chemistry, Erasmus MC, University Medical Center Rotterdam, GD Rotterdam, Netherlands; 8 Academic Center for Growth Disorders, Erasmus MC, University Medical Center Rotterdam, GD Rotterdam, Netherlands; 9 Dutch Center of Reference for Prader-Willi Syndrome, GD Rotterdam, Netherlands

**Keywords:** Prader-Willi syndrome, cardiovascular system, missed diagnosis, comorbidity, failure to rescue, health care

## Abstract

**Context:**

Prader-Willi syndrome (PWS) is a complex hypothalamic disorder, combining hyperphagia, hypotonia, intellectual disability, and pituitary hormone deficiencies. Annual mortality of patients with PWS is high (3%). In half of the patients, the cause of death is obesity related and/or of cardiopulmonary origin. Health problems leading to this increased mortality often remain undetected due to the complexity and rareness of the syndrome.

**Objective:**

To assess the prevalence of health problems in adults with PWS retrospectively.

**Patients, Design, and Setting:**

We systematically screened 115 PWS adults for undiagnosed health problems. All patients visited the multidisciplinary outpatient clinic for rare endocrine syndromes at the Erasmus University Medical Center, Rotterdam, Netherlands. We collected the results of medical questionnaires, interviews, physical examinations, biochemical measurements, polygraphy, polysomnography, and radiology.

**Main outcome measures:**

Presence or absence of endocrine and nonendocrine comorbidities in relation to living situation, body mass index, genotype, and demographic factors.

**Results:**

Seventy patients (61%) had undiagnosed health problems, while 1 in every 4 patients had multiple undiagnosed health problems simultaneously. All males and 93% of females had hypogonadism, 74% had scoliosis, 18% had hypertension, 19% had hypercholesterolemia, 17% had type 2 diabetes mellitus, and 17% had hypothyroidism. Unfavorable lifestyles were common: 22% exercised too little (according to PWS criteria) and 37% did not see a dietitian.

**Conclusions:**

Systematic screening revealed many undiagnosed health problems in PWS adults. Based on patient characteristics, we provide an algorithm for diagnostics and treatment, with the aim to prevent early complications and reduce mortality in this vulnerable patient group.

## Introduction

Prader-Willi syndrome (PWS) is a rare genetic, neuroendocrine condition caused by the absence of a normal paternal contribution to the 15q11-13 region. It is most commonly caused by a paternal deletion (65–75%) or a maternal uniparental disomy 15 ([mUPD], 20–30%). In the minority of cases, PWS is caused by an imprinting center defect (ICD, 1–3%) or a paternal chromosomal translocation (0.1%) (1, 2). The syndrome is characterized by hypotonia, behavioral challenges, typical dysmorphic features, and hypothalamic dysfunction resulting in hyperphagia, pituitary hormone deficiencies, abnormal temperature regulation, and inadequate pain registration (3–6).

Annual mortality in adults with PWS is high (3%) (7, 8) compared with 1.3% annual mortality in non-PWS adults with an intellectual disability (9). More than half of these deaths are caused by cardiopulmonary pathology (8, 10) and another 7% of deaths are directly related to obesity (8). Seventy-eight percent of deaths in patients with PWS are unexpected (11).

**Figure 1.**
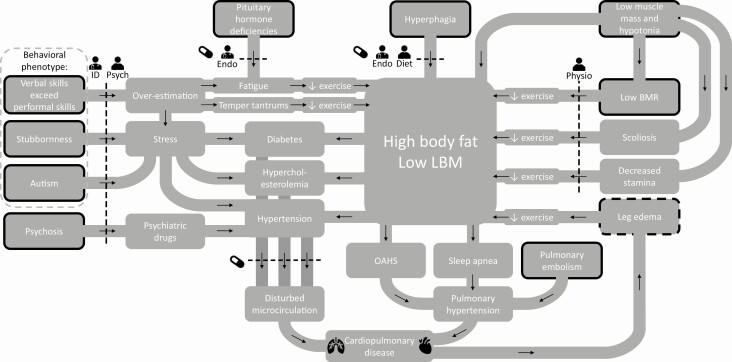
Factors contributing to cardiopulmonary disease in patients with PWS. Abbreviations: BMR, basal metabolic rate; diet ,dietitian; endo, endocrinologist; ID, physician for people with intellectual disabilities; LBM, lean body mass; OAHS, obesity associated hypoventilation syndrome; physio, physiotherapist; PWS, Prader-Willi syndrome; psych, psychologist. Legend: black arrows indicate a cause-and-effect relationship; dotted lines indicate an intervention; the stands for an intervention with medication; black borders indicate that the factor is inherent to the syndrome; dotted black border indicates that the factor is inherent to the syndrome, but can be aggravated by cardiopulmonary disease (12–39).

Multiple factors contribute to the increased risk of cardiopulmonary pathology in patients with PWS. Based on our clinical experience with more than a hundred PWS adults, we hypothesize that there is a complex interaction between obesity and behavioral, endocrine, and cardiovascular (CV) risk factors that contribute to the high prevalence of cardiopulmonary disease in patients with PWS, as shown in Fig. 1.

Obesity in patients with PWS is caused by hyperphagia (leading to a high energy intake) combined with a low energy expenditure (12, 13). This low energy expenditure is caused by low muscle mass, which is part of the syndrome. Untreated pituitary hormone deficiencies like hypogonadism, hypothyroidism, and growth hormone deficiency can affect muscle mass and function, causing a further decrease in basal metabolic rate (12, 14–21).

The total energy expenditure in adults with PWS is 20% lower than in age-matched obese adults (22). This difference in energy expenditure should be compensated by either a strict diet or by exercising for at least 1 hour a day (23). However, exercise tolerance may be low, due to hypotonia, pituitary hormone deficiencies, and (severe) vitamin D deficiency (14, 24–29). Moreover, the typical behavioral phenotype and musculoskeletal problems like scoliosis, hypotonia, and leg edema impair physical activity in adults with PWS. This results in a vicious circle of muscle weakness, exercise intolerance, and a further decrease in physical activity. The subsequent sedentary lifestyle can induce CV risk factors like hypertension, hypercholesterolemia, and type 2 diabetes mellitus (DM2) (30). Other CV risk factors often present in PWS are obesity hypoventilation syndrome and sleep apnea, which can be central sleep apnea, obstructive sleep apnea, or both. Central sleep apnea, obstructive sleep apnea, and obesity hypoventilation syndrome can lead to pulmonary hypertension (31, 32), DM2 (33), and a further increase in obesity (34) and CV risk (35–38). Lastly, the cognitive phenotype of PWS (often higher verbal comprehension skills compared with performal/reasoning skills, which can easily lead to overestimation) and autism-related behavioral challenges could induce stress. Stress can induce hypertension, another important CV risk factor (39). Moreover, psychosis is prevalent in patients with PWS, often requiring psychiatric drugs. As many psychiatric drugs have CV side effects, this can lead to a further increase in CV risk (40).

The complex interplay between somatic and psychological factors requires a syndrome-specific approach to health problems. However, as PWS is a rare disorder (5), most physicians are unfamiliar with the syndrome and its associated comorbidities. Furthermore, the PWS-specific behavioral phenotype (high pain threshold and the inability to express complaints) often leads to underdiagnosis and undertreatment. Combined doctors’ and patients’ delay can lead to medical complications and hospital admission. Timely recognition of comorbidities can reduce medical complications and associated personal and financial burdens (41).

Previous authors have reported health problems in adults with PWS (11, 42–60). However, most of them did not perform a systematic screening and only reported health problems that had already been diagnosed. As underdiagnosis is a serious problem in this patient population, the prevalences reported in these studies are most likely underestimated. Data from systematic health screenings in adults with PWS are scarce (44, 45, 47, 48, 58) and little is known about the relation between patient characteristics (living situation, presence or absence of obesity, genotype, and demographic factors) and health problems. As a consequence, there is no consensus about periodical screening.

In our reference center, we routinely perform a systematic health screening in all adults with PWS in order to detect comorbidities at an early stage. In this article, we report the prevalence of the physical health problems detected by our screening. Based on their associations with the aforementioned patient characteristics, we provide practical advice for medical screening.

## Methods

This study was approved by the Medical Ethics Committee of the Erasmus University Medical Center. In this retrospective study, we reviewed the medical files of all adults who visited the multidisciplinary outpatient clinic of the PWS reference center in the Erasmus University Medical Center, Rotterdam, Netherlands, between January 2015 and April 2020 and who underwent a routine systematic health screening. All patients that visited our outpatient clinic were already diagnosed with PWS, often years before visiting our outpatient clinic and/or during childhood. Before the launch of our multidisciplinary outpatient clinic in 2015, many Dutch adults with PWS were treated by their general practitioner or physician for people with intellectual disabilities (ID physician).

Our systematic screening consists of a structured interview, a complete physical examination, a medical questionnaire, a review of the medical records, biochemical measurements and, if indicated and feasible, additional tests such as dual energy X-ray absorptiometry (DEXA), polygraphy (PG), and polysomnography (PSG). We report the hidden health problems that were present but undetected and/or untreated until the moment of screening. Conditions that developed during later follow-up visits were not taken into account. Forty-two patients in the cohort that we describe were also mentioned in a previous study by Sinnema et al (43), who gave an overview of 102 adults with PWS and the health problems that had already been diagnosed (without systematic screening).

### Genetic diagnosis

We performed genetic testing or collected previous genetic test results from other Dutch academic hospitals to confirm the PWS diagnosis and to determine the genetic subtype.

### Medical questionnaire

As part of regular patient care, primary caregivers filled out a medical questionnaire before visiting the outpatient clinic. This questionnaire included questions on the patient’s medical history, medication, family history, symptoms of disease, physical complaints, behavioral challenges, and social aspects such as school, relationships, and living situation. The symptoms of disease, physical complaints, and behavioral challenges are rated on a 5-point Likert scale (1 = rarely or never, 2 = not often and/or not severe, 3 = quite often and/or quite severe, 4 = often and/or severe, 5 = very often and/or very severe). A score of 3 or higher was considered clinically relevant and was further explored during the visit. Mutism is defined as the absence of speech.

### Biochemical analysis

During the visit, blood samples were taken for general medical screening, including the evaluation of fat metabolism (low density lipoprotein [LDL]-cholesterol), glucose metabolism (nonfasting glucose, hemoglobin A1c), thyroid function (free T4), gonadal function (random luteinizing hormone [LH], follicle-stimulating hormone, estradiol or testosterone, sex hormone binding globulin), liver enzymes (aspartate transaminase, alanine transaminase, alkaline phosphatase, gamma glutamyl transpeptidase, total bilirubin, lactate dehydrogenase), kidney function (urea, creatinine, estimated glomerular filtration rate [eGFR]), the hematopoietic system (hemoglobin, hematocrit, mean corpuscular volume, leukocytes, thrombocytes and, in case of microcytic anemia, ferritin), and vitamin D status (25-hydroxyvitamin D). The eGFR is estimated by the Chronic Kidney Disease Epidemiology Collaboration (CKD-EPI) formula.

#### Cutoff levels.

A hypercholesterolemia diagnosis was confirmed if the patient had a nonfasting LDL-cholesterol above 4.0 mmol/L. Type 2 diabetes mellitus was defined as a repeated fasting glucose above 6.7 mmol/L (or nonfasting glucose above 11.0 nmol/L). Hemoglobin A1c was used to assess long-term glycemic control. As hypothyroidism in PWS can be both primary and central (61), hypothyroidism was defined as a free T4 below 11 pmol/L, regardless of thyroid stimulating hormone. Hypogonadism in males was defined as a morning testosterone level below 10.0 nmol/L or a random testosterone level below 10.0 nmol/L combined with clear clinical features of hypogonadism (sparse body hair, micropenis, and the absence of spontaneous morning erections). Hypogonadism in females was defined as absent, scarce, or irregular menses. The diagnosis of hypogonadism was based on both laboratory values and clinical parameters because of the effect of adipose tissue aromatase activity on estradiol and testosterone levels (62), and the fact that hypogonadism in PWS can be both primary and central (63). Due to intellectual disability in most patients, gynecological evaluation was not routinely performed. When females used oral contraceptives or estrogen replacement therapy before screening, we asked for the presence of the menstrual cycle before the start of estrogen replacement therapy.

Severe vitamin D deficiency was defined as a 25-hydroxyvitamin D level below 20 nmol/L and a mild vitamin D deficiency was defined as a 25-hydroxyvitamin D level below 50 nmol/L. When patients used cholesterol-lowering medications, oral antidiabetics, insulin, levothyroxine, or testosterone replacement therapy before the start of the screening, we requested pretreatment laboratory values to verify the diagnosis.

### Additional tests

We screened for hypertension by measuring blood pressure. If the patient’s blood pressure was above 140/90 mmHg, the measurement was repeated, and if it was still elevated, a 30-minute blood pressure measurement was performed. If the patient already used antihypertensive drugs, we requested pretreatment blood pressure values.

If risk factors for osteoporosis were present (untreated hypogonadism, family history of osteoporosis, previous fractures, untreated vitamin D deficiency, and/or corticosteroid treatment), we performed a DEXA scan to screen for osteoporosis or osteopenia. Osteoporosis was defined as a T-score (comparison of a person’s bone density with that of a healthy 30-year-old of the same sex) below -2.5, osteopenia was defined as a T-score between -1.0 and -2.5.

If there was a clinical suspicion of scoliosis (based on a gibbus deformity during physical examination), we performed an X-ray of the spine if (1) the patient was not previously diagnosed with scoliosis, (2) the patient suffered from back pain, or (3) the caregivers reported new or progressive postural abnormalities. Radiologically confirmed scoliosis was defined as a Cobb angle of 10° or more, as measured on a spinal X-ray.

The indication for sleep studies was based on the presence of clinical signs of sleep apnea: severe snoring, witnessed apneas, daytime sleepiness, morning headaches, hypertension, or waking up with shortness of breath, headaches, or panic. If indicated and feasible, we performed PG (ie, the continuous recording of nasal airflow, thoracic and abdominal movements, heart rate, and oxygen saturation during 1 night) or a PSG (ie, PG measurements and electroencephalography, electro-oculography, and electromyography). Also, before the start of growth hormone (GH) treatment, we performed a PSG to exclude sleep apnea, as untreated sleep apnea is an absolute contraindication for GH treatment.

### Data analysis

Statistical analysis was performed using R version 3.6.3. Descriptive statistics for continuous variables are reported as median and interquartile range (IQR). For dichotomous variables, we display the number of people and the percentage of total people, n (%). We used a chi-squared test to compare the prevalence of health problems between different groups based on patient characteristics: genotype, living situation, and gender. To investigate the relationship between body mass index (BMI), age, patient characteristics, and the prevalence of health problems, we used the Wilcoxon rank sum test. For the relationship between BMI, age, and living situation, we used the Kruskal-Wallis test. A chi-squared test for trend was used to compare the number of undiagnosed health problems between subgroups. To investigate the relationship between age and BMI, the Kendall rank correlation test was used. To investigate the effect of BMI and age on health problems and the number of undiagnosed health problems corrected for age and BMI respectively, logistic and ordinal regression models were used and a likelihood ratio test was performed.

### Literature review

We reviewed the literature for studies that report physical health problems in patients with PWS. Inclusion criteria were original research articles and observational studies that reported the prevalence of physical health problems in a cohort of patients with PWS of 15 years of age or older. Exclusion criteria were clinical trials, basic or translational research, case reports, case series that included less than 10 adults with PWS, articles that were not available online, articles that were not available in English, and mixed pediatric–adult articles that did not report separate prevalences for patients with PWS of 15 years of age or older. The full search strategy used is available upon request.

## Results

We included 115 (56 male and 59 female) patients. Median age was 29 years (IQR 21–40) and median BMI 29 kg/m^2^ (IQR 26–35). Baseline characteristics are shown in Table 1. The exact age at diagnosis was known for 72 patients, of which 59 patients were diagnosed during childhood. Of 115 patients, 42 underwent transition after years of medical supervision at the pediatric multidisciplinary outpatient clinic. All patients referred by the pediatrician had a personal care plan. Of the remaining 73 patients, 17 patients had been followed by an endocrinologist elsewhere during the year before the screening. A total of 46 patients had been followed by an ID physician and 14 had never visited an adult endocrinologist or ID physician before.

**Table 1.** Baseline characteristics of 115 adults with Prader-Willi syndrome

	Total, N = 115
Age in years, median [IQR]	29 [21–40]
BMI in kg/m^2^, median [IQR]	29 [26–35]
Male gender, n (%)	56 (49%)
Genetic subtype	
Deletion, n (%)	64 (56%)
mUPD, n (%)^*a*^	41 (36%)
ICD, n (%)	3 (3%)
Unknown, n (%)	7 (6%)
Growth hormone treatment	
Only during childhood, n (%)	10 (9%)
Only during adulthood, n (%)	3 (3%)
Both, n (%)	40 (35%)
Never, n (%)	62 (54%)
Current growth hormone treatment, n (%)	41 (36%)
Use of hydrocortisone	
Daily, n (%)	4 (4%)
During physical or psychological stress, n (%)	47 (41%)
Use of estrogen replacement therapy or oral contraceptives, n (%)	34/59 females (58%)
Use of levothyroxine, n (%)	17 (15%)
Living situation	
With family, n (%)	28 (24%)
In a specialized PWS group home, n (%)	23 (20%)
In a non-specialized group home, n (%)	61 (53%)
Assisted living, n (%)	3 (3%)
Scholar level	
Secondary vocational education, n (%)	6 (5%)
Prevocational secondary education, n (%)	3 (3%)
Special education, n (%)	82 (71%)
No education, n (%)	4 (4%)
Unknown, n (%)	20 (17%)
Mutism, n (%)	3 (3%)
Relationship status	
In a relationship with sexual intercourse, n (%)	8 (7%)
In a relationship without sexual intercourse, n (%)	18 (16%)
Not in a relationship, n (%)	76 (66%)
Unknown, n (%)	13 (11%)

Abbreviations: BMI, body mass index; ICD, imprinting center defect; IQR, interquartile range; mUPD, maternal uniparental disomy; PWS, Prader-Willi syndrome.

^*a*^ In 11 patients with an mUPD, the parents were not available for genetic testing. Therefore, mUPD is the most likely genotype, but an ICD could not be ruled out in these patients.

We refer to the repository (64) for the following supplementary data: baseline characteristics and health problems by living situation and genotype; health problems by BMI, age, and gender; information about lifestyle, behavior, and physical complaints; details of biochemical analysis (liver panel, kidney function, hematopoiesis, and electrolyte values); and data about sleep apnea, bone mineral density, and vitamin D deficiency.

### Health problems detected by screening

**Figure 2.**
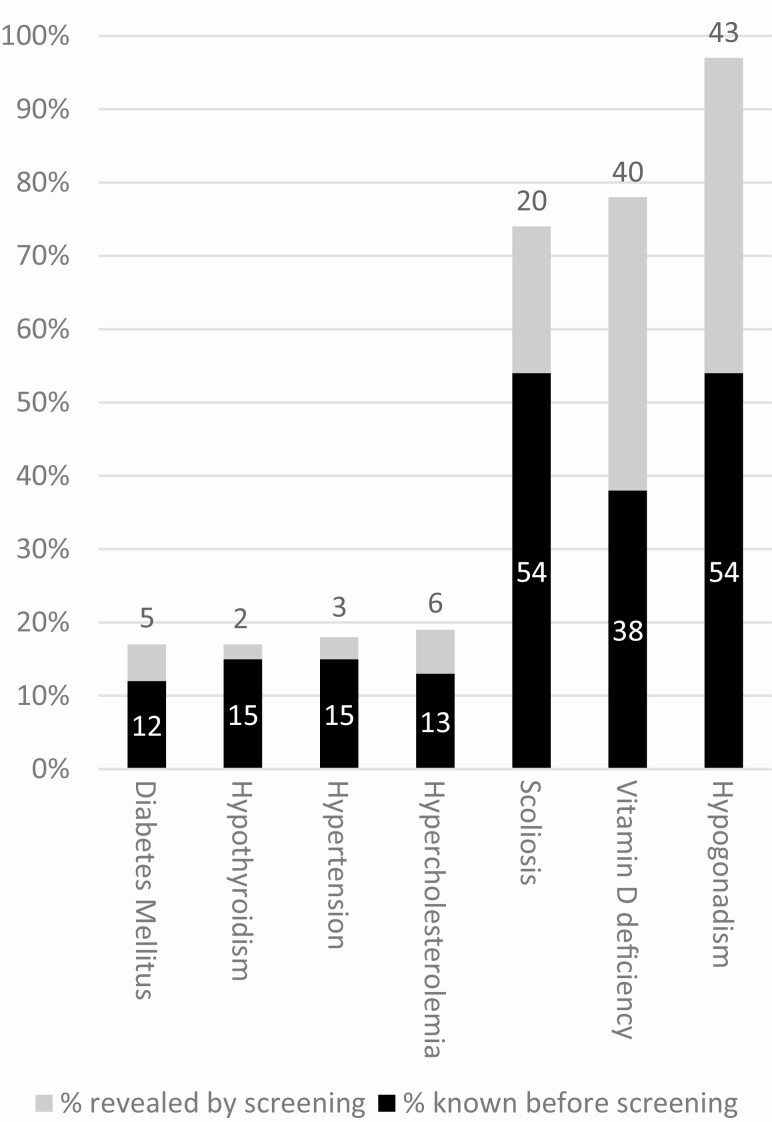
Health problems detected by systematic health screening in 115 adults with PWS. Abbreviation: PWS, Prader-Willi syndrome. Legend: black bars indicate the percentage of health problems already diagnosed before the screening; gray bars indicate the percentage of health problems that were revealed by screening.

The results of our systematic health screening are shown in Table 2 and Fig. 2. We found undetected health problems in 61% of adults with PWS. One-fourth had more than 1 undetected simultaneous health problem. The most common undetected health problem was hypogonadism, which had gone unnoticed in 52% of males and 33% of females. Other undiagnosed health problems were scoliosis (20%), hypercholesterolemia (6%), DM2 (5%), hypertension (3%), and hypothyroidism (2%). Forty-five patients underwent DEXA scans as part of medical screening. This revealed 3 new cases of osteoporosis and 8 cases of osteopenia, on top of the 9 patients already known with osteoporosis and the 22 patients with osteopenia. Two males and 1 female (all older than 30 years of age during the screening) had osteoporosis despite previous treatment for hypogonadism. Both males had received testosterone replacement therapy for more than 15 years before screening. For the female, the exact duration of estrogen replacement therapy was unknown. Nine patients were known to have sleep apnea before the screening. Nineteen patients underwent PG or PSG, of which 11 patients were diagnosed with sleep apnea.

**Table 2.** Health problems in 115 adults with PWS before and after systematic screening

	Total, N = 115			
	Before Screening	Detected by Screening	After Screening	Missing
Hypogonadism				
Male (n = 56)	26 (48%)	**+52%**	54 (100%)	2
Female (n = 59)	26 (60%)	**+33%**	40 (93%)	16^*a*^
Scoliosis	61 (54%)	**+20%**	83 (74%)^*b*^	3
Hypercholesterolemia	14 (13%)	**+6%**	22 (19%)	2
Type 2 diabetes mellitus	13 (12%)	**+5%**	19 (17%)	2
Hypertension	17 (15%)^*c*^	**+3%**	20 (18%)	3^*d*^
Hypothyroidism	17 (15%)	**+2%**	19 (17%)	0
Vitamin D deficiency	26 (38%)	**+40%**	54 (78%)	46^*e*^
Severe vitamin D deficiency			8 (13%)	55
Total undiagnosed health problems				
At least 1		**70 (61%)**		
At least 2		**28 (24%)**		
3 or more		**10 (9%)**		

Data are presented as n (% of total). In bold are the number and % of health problems detected by our systematic screening.

Abbreviation: PWS, Prader-Willi syndrome.

^*a*^ (Caregivers of) 16 female patients did not recall whether they had had a normal menstrual cycle before the start of oral contraceptives or before reaching menopausal age.

^*b*^ Twenty-eight patients had clear scoliosis at physical examination, but X-ray was not performed due to practical/behavioral issues, and 55 cases were radiologically confirmed.

^*c*^ Four patients received antihypertensive medication before screening, but the indication was unknown.

^*d*^ Blood pressure was high in 2 patients, but the measurement was not repeated due to practical/behavioral issues.

^*e*^ In 2 patients vitamin D was not measured and 44 patients used vitamin D supplementation before the screening, but it was unknown whether they had low vitamin D values before the start of vitamin D supplementation.

### Comparison of health problems between groups

#### Living situation.

 Twenty-three patients lived in a specialized PWS group home (PWS home), 61 in a nonspecialized group home (non-PWS home), 28 with family, and 3 in an assisted living facility. Patients living in non-PWS homes were significantly older (median age 36 years, IQR 28–50) than those living in PWS homes (median age 26 years, IQR 21–32) or with family (median age 19 years, IQR 19–22). Body mass index and prevalence of hypertension were significantly higher in patients living in non-PWS homes (see Table 3).

**Table 3.** Health problems according to living situation and genotype

	Missing	PWS Home^*a*^, N = 23	Non-PWS home^*b*^, N = 61	Family^*c*^, N = 28	*P*-value	Deletion, N = 64	mUPD, N = 41	*P*-value
Age, median [IQR]	0	26 [21–32]	36 [28–50]	19 [19–22]	**<0.001**	28 [21–36]	32 [21–49]	0.2
BMI, median [IQR]	0	27 [22–30]	30 [27–40]	28 [26–36]	**0.004**	31 [26–38]	29 [25–34]	0.3
Undiagnosed health problems^*d*^								
At least one		9 (39%)	44 (72%)	15 (54%)	0.16	37 (58%)	25 (61%)	0.7
At least two		2 (9%)	19 (31%)	5 (18%)		14 (22%)	12 (29%)	
Three or more		2 (9%)	4 (7%)	4 (14%)		6 (9%)	3 (7%)	
Hypogonadism								
Male (n = 56)	2	9 (100%)	28 (100%)	15 (100%)	NA^*e*^	27 (100%)	19 (100%)	NA^*e*^
Female (n = 59)	16^*f*^	10 (100%)	20 (87%)	10 (100%)	0.2	25 (93%)	14 (93%)	0.9
Hypothyroidism	0	4 (17%)	12 (20%)	3 (11%)	0.6	11 (17%)	7 (17%)	0.99
Type 2 diabetes mellitus	2	2 (9%)	13 (22%)	3 (11%)	0.2	8 (13%)	10 (24%)	0.1
Hypertension	3	0 (0%)	17 (29%)	2 (7%)	**0.002**	9 (15%)	8 (20%)	0.5
Hypercholesterolemia	2	4 (17%)	15 (25%)	2 (7%)	0.1	11 (17%)	8 (20%)	0.7
Scoliosis	3	18 (78%)	44 (76%)	19 (68%)	0.6	51 (81%)	23 (59%)	**0.02**
Vitamin D deficiency	46^*g*^	14 (88%)	22 (85%)	16 (64%)	NA^*h*^	33 (80%)	19 (76%)	NA^*h*^

Data are presented as n (% of total). *P*-values <0.05 are bold.

Abbreviations: BMI, body mass index; IQR, interquartile range; mUPD, maternal uniparental disomy; PWS, Prader-Willi syndrome.

^*a*^ Patients living in a specialized Prader-Willi syndrome group home.

^*b*^ Patients living in a non-specialized group home.

^*c*^ Patients living with family.

^*d*^ Undiagnosed health problems are hypogonadism, hypothyroidism, type 2 diabetes mellitus, hypertension, hypercholesterolemia, scoliosis, and vitamin D deficiency.

^*e*^ Not applicable, as hypogonadism is present in 100%, regardless of patient characteristics.

^*f*^ (Caregivers of) 16 female patients did not recall whether they had had a normal menstrual cycle before the start of oral contraceptives or before reaching menopausal age.

^*g*^ In 2 patients, vitamin D was not measured, and 44 patients used vitamin D supplementation before the screening, but it was unknown whether they had low vitamin D values before the start of vitamin D supplementation.

^*h*^ A *P*-value could not be calculated due to selective missings.

Patients in PWS homes exercised more than those living with family or in non-PWS homes. Patients in PWS homes all exercised at least 30 minutes a day versus 75% and 70% of those living with family or in non-PWS homes, respectively. A dietitian was involved in the care of 87% of patients living in PWS homes, 74% of those living in a non-PWS home, and 29% of those living with family.

#### Genotype.

 When comparing health problems between the 2 largest genotypic subgroups (64 patients with a deletion and 41 with mUPD), scoliosis was more frequent in patients with a deletion than in patients with an mUPD (81% vs 59%, *P* = 0.02). Other variables were not remarkably different between the genotypes (see Table 3).

#### Body mass index.

 Body mass index increased with age (*P* = 0.02). Patients with a higher BMI had a higher total number of undiagnosed health problems (*P* < 0.001) and more hypercholesterolemia (*P* = 0.01) (see Table 4). This remained significant after correction for age.

**Table 4.** Health problems according to BMI, age, and gender

	BMI <25kg/ m^2^, N = 24	BMI 25–30kg/ m^2^, N = 43	BMI >30kg/ m^2^, N = 48	*P*-value^*a*^	Corrected *P*-value for Age^*b*^	Age <25 Years, N = 43	Age 25–30 Years, N = 21	Age > 30 years, N = 51	*P*-value^*a*^	Corrrected *P*-value for BMI^*c*^	Male, N = 56	Female, N = 59	*P*-value
Age, median [IQR]	24 [20–33]	27 [20–40]	32 [25–42]	**0.02**	NA	20 [19–22]	28 [26–29]	41 [34–52]	NA	NA	31 [20–43]	28 [22–38]	0.5
BMI, median [IQR]	22 [21–23]	27 [27–29]	38 [34–46]	NA	NA	27 [23–31]	29 [26–35]	31 [27–42]	**0.02**	NA	27 [25–32]	32 [27–40]	**0.02**
Undiagnosed health problems													
At least 1	8 (33%)	24 (56%)	38 (79%)	**<0.001**	**<0.001**	19 (44%)	9 (43%)	42 (82%)	**0.001**	**0.02**	37 (66%)	33 (56%)	0.1
At least 2	2 (8%)	9 (21%)	17 (35%)	–	–	6 (14%)	4 (19%)	18 (35%)	–	–	17 (30%)	11 (19%)	
3 or more	0 (0%)	4 (9%)	6 (13%)	–	–	3 (7%)	3 (7%)	4 (8%)	–	–	7 (13%)	3 (5%)	
Hypogonadism													
Male (n = 56)	11 (100%)	27 (100%)	16 (100%)	NA	NA	20 (100%)	7 (100%)	27 (100%)	NA	NA	54 (100%)	40 (93%)	NA
Female (n = 59)	6 (100%)	12 (92%)	22 (92%)	0.9	0.3	18 (100%)	9 (90%)	13 (87%)	0.2	0.1	–	–	NA
Hypothyroidism	5 (21%)	7 (16%)	7 (15%)	0.5	0.8	10 (23%)	5 (24%)	4 (8%)	0.2	0.4	5 (9%)	14 (24%)	**0.03**
Type 2 diabetes mellitus	2 (8%)	7 (17%)	10 (21%)	0.2	0.6	2 (5%)	2 (10%)	15 (31%)	**<0.001**	**0.001**	13 (24%)	6 (10%)	0.06
Hypertension	3 (13%)	6 (15%)	11 (23%)	0.4	0.7	3 (7%)	1 (5%)	16 (31%)	**<0.001**	**<0.001**	9 (17%)	11 (19%)	0.8
Hypercholesterolemia	4 (17%)	4 (10%)	14 (30%)	**0.01**	**0.01**	3 (7%)	2 (10%)	17 (35%)	**0.002**	**0.01**	10 (18%)	12 (21%)	0.7
Scoliosis	12 (79%)	30 (71%)	34 (74%)	0.3	0.2	30 (70%)	19 (90%)	34 (71%)	0.9	0.5	42 (76%)	41 (72%)	0.6
Vitamin D deficiency	12 (75%)	20 (77%)	22 (81%)	NA^*d*^	NA^*d*^	27 (69%)	10 (91%)	17 (89%)	NA^*d*^	NA^*d*^	25 (83%)	29 (74%)	NA^*d*^

*P*-values <0.05 are bold.

Abbreviations: BMI, body mass index; IQR, interquartile range; NA, not available.

^*a*^ *P*-value calculated with BMI and age as continuous variables.

^*b*^ *P*-value corrected for age using regression models.

^*c*^ *P*-value corrected for BMI using regression models.

^*d*^A *P*-value could not be calculated due to selective missings.

#### Age.

 Older patients had a higher prevalence of DM2 (*P* < 0.001), hypertension (*P* < 0.001), and hypercholesterolemia (*P* < 0.002), and a higher total number of undiagnosed health problems (*P* = 0.001) (see Table 4). This remained significant after correction for BMI.

#### Gender.

 Body mass index was significantly higher in females than in males (*P* = 0.02). Hypothyroidism was more prevalent in females than in males (24% vs 9%, *P* = 0.03) (see Table 4).

#### Age and BMI.

Thirteen patients had BMI < 25kg/m^2^ and age < 25 years. None of these patients had DM2, 1 patient had hypertension, and 2 had hypercholesterolemia (of which 1 case was undiagnosed before screening).

### Fatigue and daytime sleepiness

Fatigue and daytime sleepiness were common. One-third of the patients (40/115) had clinically relevant daytime sleepiness (score of 3 or higher on a 5-point Likert scale). All of these 40 patients had either untreated vitamin D deficiency, untreated male hypogonadism (Table 5), or another treatable cause such as sleep apnea, narcolepsia, nycturia, or use of drugs that can cause sleepiness (antiepileptic drugs, antipsychotics, benzodiazepines, tricyclic antidepressants, or antihistamines). Daytime sleepiness was present in 62% of the patients with untreated vitamin D deficiency versus 36% of the patients with normal vitamin D levels (*P* = 0.02). It was also related to the severity of the deficieny: daytime sleepiness was present in 80% of patients with untreated severe vitamin D defiency, 57% of patients with untreated mild vitamin D defiency, and 36% of patients with normal vitamin D levels.

**Table 5.** Fatigue and daytime sleepiness and potential underlying causes

	Difficulty Sleeping		*P*-value	Nycturia		*P*-value	Snoring		*P*-value	Male Hypogonadism		*P*-value	Hypothyroidism		*P*-value	Untreated Vitamin D Deficiency		*P*-value
	-	+		-	+		-	+		T^*a*^	UT^*b*^		-	+		-	+	
**N**	84	9		66	28		64	32		24	31		96	19		85	28	
Fatigue	18 (22%)	4 (44%)	0.1	12 (18%)	11 (41%)	**0.03**	11 (18%)	12 (41%)	**0.02**	4 (19%)	7 (28%)	0.5	18 (23%)	5 (31%)	0.5	15 (22%)	8 (32%)	0.3
Daytime sleepiness	32 (38%)	8 (89%)	**0.003**	23 (35%)	17 (61%)	**0.02**	18 (29%)	22 (71%)	**<0.001**	7 (33%)	17 (65%)	**0.03**	35 (44%)	6 (35%)	0.5	25 (36%)	16 (62%)	**0.02**

Physical complaints were scored on a 5-point Likert scale. A score of 3 or higher was seen as clinically relevant (“+”), a score below 3 was seen as not clinically relevant (“-“).

Abbreviations: T, treated; UT, untreated.

^*a*^Treated with testosterone replacement therapy.

^*b*^Untreated hypogonadism.

### Biochemical analysis

Liver panel was normal in most patients. However, 19 patients had alkaline phosphatase levels above the upper limit of normal. The vast majority of them had potential underlying causes such as vitamin D deficiency (63%) and/or obesity (58%). Normocytic anemia was common in males, but not in females. There were no cases of micro- or macrocytic anemia. Of the 17 males with anemia, 13 (76%) had untreated hypogonadism. Creatinine levels were generally low: 35 males (63%) and 28 females (47%) had creatinine levels below the lower limit of normal, and this was independent of BMI and age. Of all males, 95% had creatinine levels between 46 and 93µmol/L and eGFR levels between 93 and 149ml/min/1.73m^2^. Of all females, 95% had creatinine levels between 37 and 76 µmol/L and eGFR levels between 98 and 140 ml/min/1.73m^2^.

### Literature review

We found 21 publications reporting 1 or more of the following health problems in PWS: hypogonadism, hypothyroidism, DM2, hypertension, hypercholesterolemia, scoliosis, vitamin D deficiency, sleep apnea, or osteoporosis/osteopenia. Outcomes of these studies are summarized in Tables 6 and 7. None of the papers reported the prevalence of vitamin D deficiency in PWS.

**Table 6.** Patient characterististics of cohorts assessed by previous studies

	Article	N^*a*^	Country	Data Collection	Age Range (years)^*a*^	Genotype (deletion/ mUPD/ICD/ translocation)	Sex	Mean BMI (kg/m^2^)	Previous GH Treatment (%)
**Papers without systematic screening**	Laurance et al (1981) (51)	24	United Kingdom	NA	15–41	NA	13 M, 11 F	NA	NA
	Greenswag (1987) (52)	232	United States of America	Q	16–64	NA	115 M, 117 F	34^*b*^	NA
	Partch et al (2000) (46)	19	Germany	MR, I, PE	18–34	NA	7 M, 12 F	46	NA
	Marzullo et al (2005) (50)	13	Italy	MR	18–NA mean ± SD: 27 ± 1	85%/15%/-/-	7 M, 6 F	46	38%
	Butler et al (2002) (53)	58	United Kingdom	I and MR	18–46	NA	32 M, 26 F	35	13%
	Thomson et al (2006) (42)	30	Australia	State health data sets	15–48	44%/10%/-/- (54% NA)^*c*^	23 M, 23 F^*c*^	NA	NA
	Sinnema et al (2011) (43)	102	Netherlands	I and MR	18–66	54%/43%/3%/-	49 M, 53 F	32	13%
	Grugni et al (2013) (49)	108	Italy	MR and PE	18–43	68%/25%/-/2% (6% NA)	47 M, 61 F	Median in nonobese: 26	NA
								Median in obese: 45	
	Proffitt et al (2019) (11)	2029	United States of America	Q	0–84	42%/19%/2%/-^*c*^ (37% NA)	934 M, 1000F^*c*^	Living: 29^*c*^	56%^*c*^
								Deceased: 52^*c,d*^	
**Papers with systematic screening**	Hertz et al (1993) (54)	15	United States of America	MR after SS	18–47	47%/-/-/- (53% NA)	7 M, 8 F	38	8%^*c*^
	Richards et al (1994) (55)	14	United Kingdom	SS	16–39	NA	9 M, 5 F	30	NA
	Höybye et al (2002) (47)	19	Sweden	SS	17–37	NA	10 M, 9 F	36	0%
	Eldar-Geva et al (2009) (56)	10	Israel	SS	15–32	50%/40%/10%/-	10 F	36	0%
	Nakamura et al (2009) (57)	34	Japan	MR after SS	16–51	79%/NA/NA/NA^*c*^	NA	NA	NA
	Van Nieuwpoort et al (2011) (48) & Van Nieuwpoort et al (2018) (58)	15	Netherlands	SS	19–43	93%/7%/-/-	4 M, 11 F	Median: 28	27%^*e*^
	Laurier et al (2015) (44)	154	France	MR after SS	16–54	66%/16%/2%/2% (15% NA)	68 M, 86 F	42	24%
	Coupaye et al (2016) (45)	73	France	MR after SS	16–58	64%/36%/-/-	35 M, 38 F	Deletion: 41 mUPD: 35	36%
	Fintini et al (2016) (59)	145	Italy	MR after SS	18–50	66%/32%/-/-^*c*^ (2% NA)	59 M, 86 F	41	15%
	Ghergan et al (2017) (60)	60	France	SS	16–54	65%/28%/2%/- (5% NA)	26 M, 34 F	39	27%
	Pellikaan et al (2020) (64)	115	Netherlands	MR after SS	18–72	56%/36%/3%/- (6% NA)	56 M, 59 F	32	46%

Systematic screening is defined as a systematic analysis of all outcomes in which all patients are subject to I, PE, Q, laboratory analysis and/or additional testing in order to detect or exclude each diagnosis. *P*-values <0.05 are bold.

Abbreviations: BMI, body mass index; F, females; GH, growth hormone; I, interviews; ICD, imprinting center defect, M, males; MR, medical records; mUPD, maternal uniparental disomy; deletion, paternal deletion; NA, not available; PE, physical examination; Q, questionnaires; SS, systematic screening.

^*a*^When a subgroup analysis was performed, the N and age range for the adult group (15 years or older) is reported.

^*b*^Approximation based on mean weight and height in the total population.

^*c*^Percentages or values based on the whole cohort of children and adults, as the values for the adult group alone are unknown.

^*d*^BMI level at greatest weight.

^*e*^ Patients with current GH treatment were excluded.

**Table 7.** Health problems assessed by previous studies

Article	Hypertension (%)	Type 2 Diabetes Mellitus (%)	Hypercholesterolemia (%)	Sleep Apnea (%)	Scoliosis (%)	Osteoporosis (%)	Hypogonadism (%)	Hypothyroidism (%)
Laurance et al (1981) (51)	–	17%	–	–	58%	–	92% F (MC)	–
Greenswag (1987) (52)	17%	19%	–	–	± 50%	–	94% F (MC)	–
Partsch et al (2000) (46)	16%	16%	37%	58%	37%	–	100% M / 100% F (MC)	–
Marzullo et al (2005) (50)	23%	8%	–	–	–	–	100% F (MC)	–
Butler et al (2002) (53)	13%	24%	–	–	34%	2%	–	–
Thomson et al (2006) (42)	–	13%	–	–	37%	3%	58% F^*a*^, 100% F (MC)	–
Sinnema et al (2011) (43)	9%	17%	–	10%	56%	16%	91% F (MC)	9%
Grugni et al (2013) (49)	48%	21%	–	–	–	–	–	5%
Proffitt et al (2019) (11)	–	11%^*b*^	–	45%^*b*^	33%^*b*^	9%^*b*^	–	9%^*b*^
Hertz et al (1993) (54)	–	–	–	7%	–	–	–	–
Richards et al (1994) (55)	–	29%^*c*^	–	86%	–	–	–	–
Höybye et al (2002) (47)	–	5%	^*d*^	–	–	Osteoporosis: 21% Osteopenia: 58%	63% (LM)	0%
Eldar-Geva et al (2009) (56)	–	10%^*c*^	–	–	–	–	40% F (LM), 100% F (MC)	–
Nakamura et al (2009) (57)	–	–	–	–	44%	–	–	–
Van Nieuwpoort et al (2011) (48) & Van Nieuwpoort et al (2018) (58)	–	7%	–	–	–	Osteoporosis: 13% Osteopenia: 40%	100% M / 82% F (MC)	13%
Laurier et al (2015) (44)	25%	25%	^*e*^	35%	75%	–	–	26%
Coupaye et al (2016) (45)	16%	19%	10%	–	78%	–	96% (LM + RT)	26%
Fintini et al (2016) (59)	–	21%	–	–	–	–	–	–
Ghergan et al (2017) (60)	22%^*c*^	25%^*c*^	^*f*^	23%	–	–	–	25%^*c*^
Pellikaan et al (2020) (64)	18%	17%	19%	17%–93%^*g*^	74%	Osteoporosis: 10%–44%^*g*^ Osteopenia: 26%–60%^*g*^	100% M / 93% F (MC)	17%

Abbreviations: F, females; LM, laboratory measurements (diagnosis of hypogonadism was based on LH, FSH and/or estrogen); M, males; MC, menstrual cycle (diagnosis of hypogonadism was based on amenorrhea or oligomenorrhea); RT, replacement therapy (diagnosis of hypogonadism was based on use of estrogen).

^*a*^58% hypogonadism in females was reported; however, the method of evaluation was not described.

^*b*^Percentages or values based on the whole cohort of children and adults, as the values for the adult group alone are unknown.

^*c*^Reported, but to our knowledge not based on systematic screening.

^*d*^Total cholesterol was less than 5 mmol/liter in 16/19 patients, and 7 patients had LDL cholesterol above 3 mmol/liter, but the highest level found was 4.2 mmol/liter.

^*e*^Hyperlipidemia in 10% (hypercholesterolemia was not described).

^*f*^Dyslipidemia in 54% (hypercholesterolemia was not described).

^*g*^As polygraphy, polysomnography, and DEXA scans are not always indicated or feasible, we had many missing values for sleep apnea and osteoporosis. As the missing values were not random, we were only able to provide ranges for the prevalence of sleep apnea and osteoporosis.

### Algorithm for diagnostics and treatment

**Figure 3.**
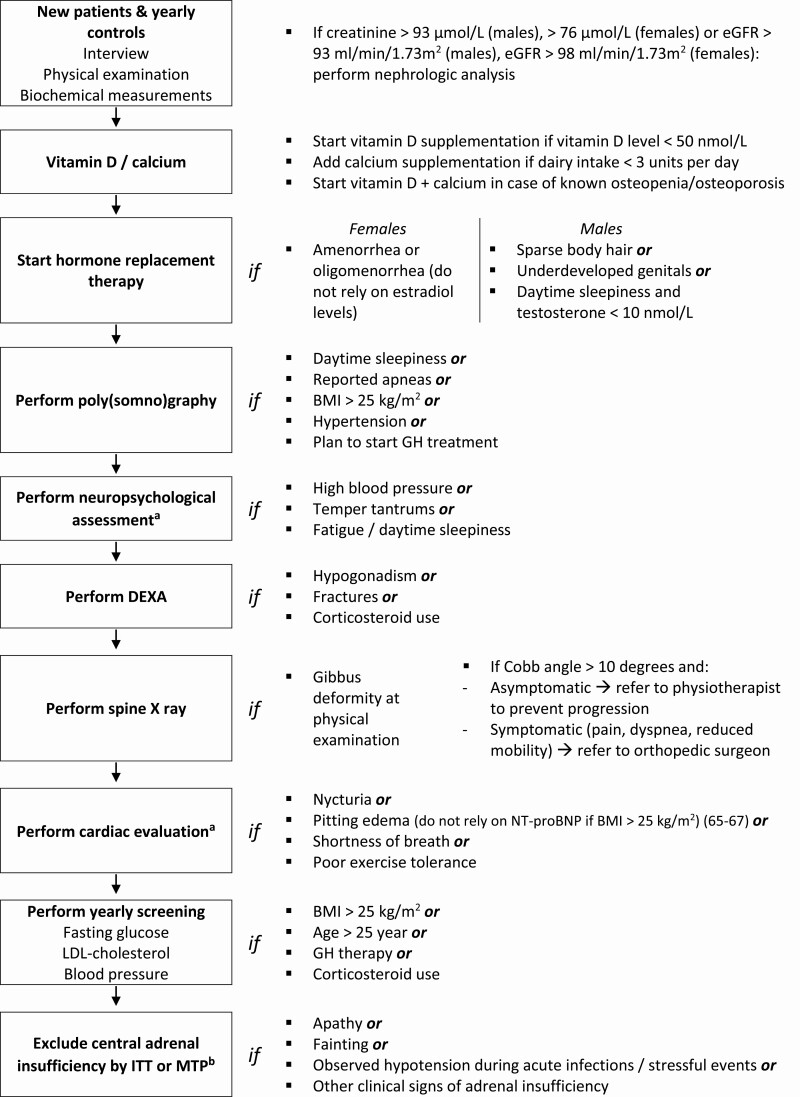
Algorithm for diagnostics and treatment in adults with PWS. Abbreviations: BMI, body mass index; DEXA, dual energy X-ray absorptiometry; FSH, follicle-stimulating hormone; FT4, free thyroxin; HbA1c, hemoglobin A1c; ITT, insulin tolerance test; LDL, low density lipoprotein; LH, luteinizing hormone; MTP, metyrapone test; PWS, Prader-Willi syndrome; SHBG, sex hormone binding globulin. ^*a*^Recommendation based on expert opinion and literature review (65–67). ^*b*^Based on previously published data (68).

Based on our analysis of patients data and the literature review, we defined diagnostic and therapeutic recommendations, presented in the algorithm in Fig. 3.

## Discussion

We found a large number of undetected health problems among adults with PWS during our systematic health screening. To our knowledge, we are the first to translate this into an evidence-based algorithm for screening and treatment of adults with PWS. We hypothesize that the high prevalence of undiagnosed health problems is the result of most physicians’ unfamiliarity with the syndrome, in combination with the complex PWS-specific behavioral phenotype.

### Previous studies

Previous studies have reported prevalences of health problems in adults with PWS. However, most studies (11, 42, 43, 46, 49–53) did not perform a systematic screening. Of the 4 studies that performed a systematic health screening (44, 45, 47, 48, 58), only 2 (Laurier et al (44) and Coupaye et al (45)) included a substantial number of (over 20) patients. Six studies (54–57, 59, 60) performed a systematic screening, but focused on only 1 health problem of interest. Compared to the systematic screening described by Laurier et al (44) and Coupaye et al (45), we found a lower prevalence of DM2, scoliosis, and hypothyroidism. The difference in the prevalence of DM2 could be partly explained by the large difference in BMI, which was much lower in our cohort than in the French cohorts (Table 7). Moreover, the patients in our cohort had more often been treated with GH during childhood. Although GH treatment may have a short-term negative effect on glucose homeostasis due to increased insulin resistance, GH treatment also improves body composition and exercise tolerance, which has positive effects on glucose metabolism in the long term (69–71).

### Prader-Willi syndrome homes and non-PWS homes

Patients in specialized PWS homes had a lower BMI and a lower frequency of hypertension than patients living in non-PWS homes. This could probably be largely explained by the age difference between the groups. Another contributing factor could be that patients in a specialized PWS home are subject to strict supervision from trained personnel. In PWS homes, food is kept out of sight and food storages are locked. According to caregivers, this greatly reduces the stress and conflicts caused by food-seeking behavior. We hypothesize it might even prevent stress-related hypertension. The fact that all patients living in PWS homes received portion-controlled meals (as determined by a dietitian) and often exercised under supervision probably explained part of the difference in the BMI between patients living in PWS homes and those living in non-PWS homes.

### Fatigue and daytime sleepiness

Fatigue and daytime sleepiness were very common problems among adults with PWS. According to caregivers, these complaints often prevented them from taking part in day trips and physical activities. Daytime sleepiness is usually attributed to a lack of hypothalamic arousal and is regarded as a problem that is inherent to the syndrome. However, when we looked in more detail, all patients with clinically relevant fatigue or daytime sleepiness had treatable underlying problems such as sleep apnea, narcolepsia, nycturia, vitamin D deficiency, untreated male hypogonadism, or use of drugs that can cause sleepiness. Although we could not perform a randomized controlled trial to assess whether treating these underlying problems resolved the complaints, our clinical experience is that the majority of the patients reported less fatigue after treatment of the underlying cause. Also, caregivers reported that these patients were more actively participating in daily activities. This indicates that daytime sleepiness is not necessarily just “part of the syndrome”, but could be the symptom of an underlying, treatable problem. Treating the underlying cause is important to reduce daytime sleepiness and increase physical activity.

### Vitamin D deficiency and hypogonadism

Both vitamin D deficiency and hypogonadism are frequently present in adults with PWS. Low levels of vitamin D and testosterone are often attributed to obesity, as vitamin D is fat-soluble and testosterone production can be diminished by increased estradiol levels due to adipose tissue aromatase activity. However, lean male patients also had hypogonadism and vitamin D defiency. Although there is no consensus on the clinical effects of vitamin D (25, 72–74), we found a clear relation between (the severity of) vitamin D deficiency and daytime sleepiness. Although the cause of daytime sleepiness in this complex patient population is likely to be multifactorial, we believe that prescribing vitamin D may be beneficial for all PWS adults with vitamin D deficiency. The high prevalence of osteoporosis and osteopenia in adults with PWS combined with the fact that vitamin D knows little side effects (75) are additional arguments for treatment. Therefore, we recommend prescribing vitamin D supplementation in all adults with PWS with a vitamin D level below 50 ng/mL.

### Creatinine levels

Creatinine levels were low in the majority of patients, regardless of sex and BMI. This indicates that normal creatinine levels in patients with PWS are lower than in healthy controls, which is explained by their low muscle mass (13). Therefore, in PWS patients, presence of high-normal creatinine levels might actually indicate impaired renal function. We recommend to adjust the reference values with –24% for males and –18% for females. In our hospital, the PWS-specific reference range for creatinine is 46 to 93 µmol/L (compared with 65–115 µmol/L for non-PWS adult males) and 37 to 76 µmol/L for females (compared with 55–90 µmol/L for non-PWS adult females). For the same reasons, we propose using PWS-specific reference values for eGFR of >98 ml/min/1.73m^2^ for PWS adult males and >93 ml/min/1.73m^2^ for PWS adult females.

### Adrenal insufficiency

This is rare in adults with PWS (68). However, in cases of clinical signs of hypocortisolism, we recommend assessing the hypothalamic-pituitary-adrenal axis using the metyrapone test or, in the absence of contraindications, the insulin tolerance test (see Fig. 3).

### Strengths and limitations

Like every study, our study has strengths and limitations. The strengths of our study are the large sample size (considering the fact that PWS is a rare syndrome), its focus on adults, and the fact that we investigated health problems in relation to living situation, BMI, genotype, and demographic factors, thus making our study a powerful source of new information. Limitations may include selection bias (due to selective referral to our specialized facility) and survival bias. Moreover, we have many missing values for osteoporosis and sleep apnea. These additional tests were not always performed because they were either not indicated or impossible to perform due to behavioral issues. Therefore, these results should be interpreted with caution.

Conclusion

We found undetected health problems in 61% of adults with PWS. On top of this, one-third of the patients had clinically relevant fatigue or daytime sleepiness which, according to caregivers, prevented them from taking part in physical activities. Although daytime sleepiness is usually considered just “part of the syndrome,” all of these patients turned out to have treatable causes such as sleep apnea, narcolepsia, nycturia, vitamin D deficiency, untreated male hypogonadism, or use of drugs that can cause sleepiness. Therefore, fatigue and daytime sleepiness should be considered not just “part of the syndrome,” but the symptom of an underlying health problem. We recommend exploring and treating these underlying causes in order to optimize physical activity and prevent obesity-related cardiopulmonary problems. We provide an algorithm for diagnostics and treatment, taking into account PWS-specific pitfalls like falsely low creatinine levels and false-normal cardiac markers. Use of the algorithm will optimize the mental and physical health of adults with PWS. This will improve exercise tolerance and reduce the personal and financial burden of cardiopulmonary complications in this vulnerable patient group.

## Data Availability

The datasets generated during and/or analyzed during the current study are not publicly available but are available from the corresponding author on reasonable request.

## Abbreviations

ALAT: alanine transaminase
ALP: alkaline phosphatase
ASAT: aspartate transaminase
BMI: body mass index
CV: cardiovascular
DEXA: dual energy x-ray absorptiometry
DM2: type 2 diabetes mellitus
eGFR: estimated glomerular filtration rate
FSH: follicle-stimulating hormone
FT4: free thyroxin
GGT: gamma glutamyl transpeptidase
GH: growth hormone
ICD: imprinting center defect
IQR: interquartile range
LDH: lactate dehydrogenase
LDL: low-density lipoprotein
LH: luteinizing hormone
mUPD: maternal uniparental disomy
PG: polygraphy
PSG: polysomnography
PWS: Prader-Willi syndrome
SHBG: sex hormone binding globulin

## Additional Information

***Disclosure Summary:*** The authors have nothing to disclose.
